# Vitamin D deficiency as a risk factor for dementia and Alzheimer’s disease: an updated meta-analysis

**DOI:** 10.1186/s12883-019-1500-6

**Published:** 2019-11-13

**Authors:** Bingyan Chai, Fulin Gao, Ruipeng Wu, Tong Dong, Cheng Gu, Qiaoran Lin, Yi Zhang

**Affiliations:** 1 Department of Neurology,Gansu Provincial People’s Hospital, No.204, Donggang West Road, Lanzhou, 730000 Gansu China; 2 School of Clinical Medicine, Gansu university of Traditional Chinese medicine, No. 35 of Dingxi East Road, Lanzhou City, Gansu Province 730000 People’s Republic of China

**Keywords:** Alzheimer’s disease, Dementia, Vitamin D deficiency, Meta-analysis

## Abstract

**Background:**

We aimed to comprehensively explore the associations between serum 25(OH)D deficiency and risk of dementia and Alzheimer’s disease(AD).

**Methods:**

We systematically searched Pubmed, the Cochrane Library, Embase and the reference lists of pertinent review articles for relevant articles published from database inception up until January 2019. Pooled hazard ratios (HRs) and 95% confidence intervals (CIs) were calculated with random effects models using the Stata 12.0 statistical software package.

**Results:**

Twelve prospective cohort studies and four cross-sectional studies were included in this meta-analysis. The pooled HRs of dementia and AD, respectively, were 1.32 (95%CI: 1.16, 1.52) and 1.34 (95%CI: 1.13, 1.60) for vitamin D deficiency (< 20 ng/ml). In the subgroup analyses, the pooled HRs of dementia and AD, respectively, were 1.48 (95%CI: 1.19, 1.85) and 1.51 (95%CI: 1.04, 2.18) for moderate vitamin D deficiency (10–20 ng/ml) and 1.20 (95%CI: 0.99, 1.44) and 1.36 (95%CI: 1.01, 1.84) for severe vitamin D deficiency (< 10 ng/ml).

**Conclusion:**

There are significant associations between vitamin D deficiency and both dementia and AD. There are stronger associations between severe vitamin D deficiency (< 10 ng/ml) and both dementia and AD compared to moderate vitamin D deficiency (10–20 ng/ml).

## Background

Dementia is one of the leading causes of death and disability. Currently, approximately 48 million people around the world are suffering from dementia and this number is expected to increase 2.73 times by 2050 [1]. Almost 10 million people progress to dementia each year; the median age of diagnosis is about 80 years of age [2]. This places an enormous burden on the health system, with AD care costing about $81.8 billion each year worldwide [3]. AD is the most common type of dementia, accounting for 75% of all dementia cases [4]. Dementia is an incurable neurodegenerative disease of unknown cause. As such, many researchers have focused on exploring relevant preventive interventions to delay the development of dementia. Several relevant risk factors have been identified; however, the evidence for these is varied and unconvincing. In addition to other potentially modifiable risk factors for dementia, such as being overweight, smoking, diabetes mellitus, hypertension, hypercholesterolemia, and cardiovascular diseases, a potential prognostic role of vitamin D deficiency has been proposed [5]. Due to the role of vitamin D in neurotrophy, neurotransmission, neuroprotection, and neuroplasticity, it has been suggested that vitamin D deficiency may play a key role in the progression of dementia and AD [5]. Some researchers refer to vitamin D as the “forgotten neurosteroid” [6]. Organisms primarily obtain vitamin D through food intake and skin synthesis. A published clinical study estimated that there are 1 billion people worldwide with vitamin D deficiency due to a lack of exposure to sunlight [7]. In addition, people residing in regions with less sunlight need to consume more foods rich in vitamin D [8]. 7-DHC in the skin is irradiated by ultraviolet rays (solar ultraviolet B radiation of 290–315 nm) and is processed by the liver to produce 25(OH)D_3_, which is a stable storage and assay form of vitamin D in the body [9]. Kidney-treated 1,25-(OH)_2_D_3_ is the only active metabolite of vitamin D in organisms [9]. Enzymes involved in the synthesis and elimination of 1,25-(OH)_2_D_3_ are expressed in brain regions such as the thalamus, hippocampus, and basal ganglia, suggesting that vitamin D has both autocrine and paracrine pathways in the central nervous system [10, 11]. In addition, studies have shown that gene polymorphisms of vitamin D are associated with AD susceptibility [12]. To date, several systematic reviews and clinical trials have discussed the relationship between vitamin D deficiency and cognitive decline, but the conclusions of these studies are contradictory. A recent meta-analysis of five cohort studies showed that adequate vitamin D was associated with lower risk of dementia and AD, and the researchers excluded cross-sectional studies, with possible choice bias and publication bias. A recent meta-analysis of five cohort studies showed that adequate vitamin D was associated with lower risk of dementia and AD; the authors of this study excluded cross-sectional studies and those with possible choice bias and publication bias [13]. Another meta-analysis of 18,974 adults reported that severe vitamin D deficiency (< 10 ng/ml) increased the risk of dementia by 54% [14]. However, a recent prospective cohort study of 13,044 participants in the Middle East reported that lower concentrations of vitamin D measured during middle age were not significantly associated with faster cognitive decline during the 20-year follow-up period [15]. Another systematic review also failed to find a significant correlation between cognitive decline and plasma 25-(OH)D concentration [16]. There are several limitations of the existing literature. Previous systematic reviews are now outdated, with the many recently published studies not included in the available meta-analyses, and most published studies had insufficient data for subgroup analyses and exploration of correlations. Further, few previous meta-analyses have examined the associations between moderate vitamin D deficiency (10–20 ng/ml) and both dementia and AD. Further, the inconsistency in the results of meta-analyses may be due to differences in literature search strategies, inclusion criteria, statistical analyses, and adjustments for confounding factors. Vitamin D deficiency is common worldwide among all age subgroups. A cross-sectional analysis using Pearson’s correlations found that low vitamin D levels in the elderly and also in young people (30–60 years old) were correlated with significant declines in cognitive ability [17]. However, there has been no systematic evaluation of whether vitamin D deficiency (< 20 ng/ml) is associated with risk of development of dementia and AD. Such information would be significant for preventing the development of dementia. Therefore, the aim of this study was to estimate the associations between vitamin D deficiency and the risk of developing dementia and AD, respectively.

## Methods

### Search strategy

A systematic literature search of Pubmed, the Cochrane Library, and Embase was conducted to identify relevant studies published from database inception until 1 January 2019. The literature search strategy was based on the PICO principles [18]. The search included combinations of keywords relevant to “vitamin D” or “25(OH)D”, “Alzheimer’s disease” or “dementia”, and so forth. All terms within each set were combined with a Boolean operator. Boolean operations, also known as logical operations, are mathematical methods that can be applied to a literature search strategy; they are simple terms such as “OR” or “END” that are used to connect key terms. The reference lists of all retrieved articles were also manually searched to identify relevant studies that were missed by the search strategy. Only studies published in English were eligible for inclusion. All considered studies were imported into a reference management software (Endnote X·8·0·0) and duplicate publications were deleted.

### Inclusion criteria and study selection

The diagnostic criteria for AD referred to the National Institute of Neurological and Communicative Disorders and Stroke and the Alzheimer Diseases and Related Disorders Associations (NINCDS-ADRDA) revised in the United States in 2011. The selection criteria included: (1) human subjects, (2) aged over 18 years, (3) observational (cross-sectional, case-control, longitudinal) or interventional designs with a control group, (4) blood measurement of 25(OH)D, (5) effective neuropsychological tests, (6) reported risk estimates (relative risk or hazard ratios (HRs) and corresponding 95% confidence intervals (CIs) for dementia or AD in relation to each category of serum 25(OH)D, and (7) reported dementia or AD incidence at follow-up. The exclusion criteria included: (1) studies with incomplete data (relative risk or hazard ratios for dementia not reported or study published only as an abstract), (2) studies examining other psychological, metabolic, or neurological conditions, and (3) participants with a diagnosis of dementia or cognitive disorder at baseline. Two independent authors (FL-G, RP-W) screened the titles and abstracts of all retrieved studies against pre-specified criteria. Disputes about inclusion or exclusion of a study were solved by discussion to reach consensus or by involving a third reviewer.

### Data extraction and assessment of study quality

Pre-designed, standardized data extraction forms were developed to capture pertinent information from each included study. The collected information included: the first author’s name, year of publication, country, study design, mean age, follow-up duration, gender, adjusted factors, assessment method for serum 25(OH)D, sample size, and reported risk estimates and 95% CIs for dementia and AD across different categories of serum 25(OH)D. If a study did not provide enough information to extract the corresponding data, the authors were contacted to try and obtain the required additional information. Two authors independently extracted the data from each study and compared the data for consistency. Risk of bias was assessed using the Cochrane risk of bias tool and NOS. Any discrepancies were resolved by group discussion to reach consensus.

### Data synthesis and statistical analysis

HRs and 95% CIs were considered as measures of effect size for all studies. All statistical analyses were conducted using the Stata 12.0 software package. We followed the recommendations of the Institute of Medicine and divided serum vitamin 25(OH)D into three categories: < 10 ng/ml (severe deficiency), 10–20 ng/ml (moderate deficiency), and ≥ 20 ng/ml (non-deficient or sufficient, as the reference category). Study heterogeneity was assessed by *I*^*2*^ estimations. According to the Cochrane review group criteria, study heterogeneity was divided into three levels: low heterogeneity (*I*^*2*^ < 25%), moderate heterogeneity (*I*^*2*^ 25–50%), and high heterogeneity (*I*^*2*^ > 50%). A fixed effects model was used when no statistical heterogeneity was detected; otherwise, a random effects model was used. If there is no heterogeneity among the included studies, the results of the fixed effect model and the random effects model are consistent. Relevant subgroup analyses considered APOE genes and study types. Publication bias was evaluated by Begg’s test. If the funnel plot was visually symmetric or *P* > 0.05, there was considered to be no publication bias. In addition, we also explored the association between risk of dementia or AD and follow-up time using meta-regression. Correlation coefficients were expressed as β. Sensitivity analysis was also performed; each study was eliminated in turn to examine the stability of the results. Significance was set at a *P* value less than 0.05.

## Results

### Study characteristics

**Fig. 1:**
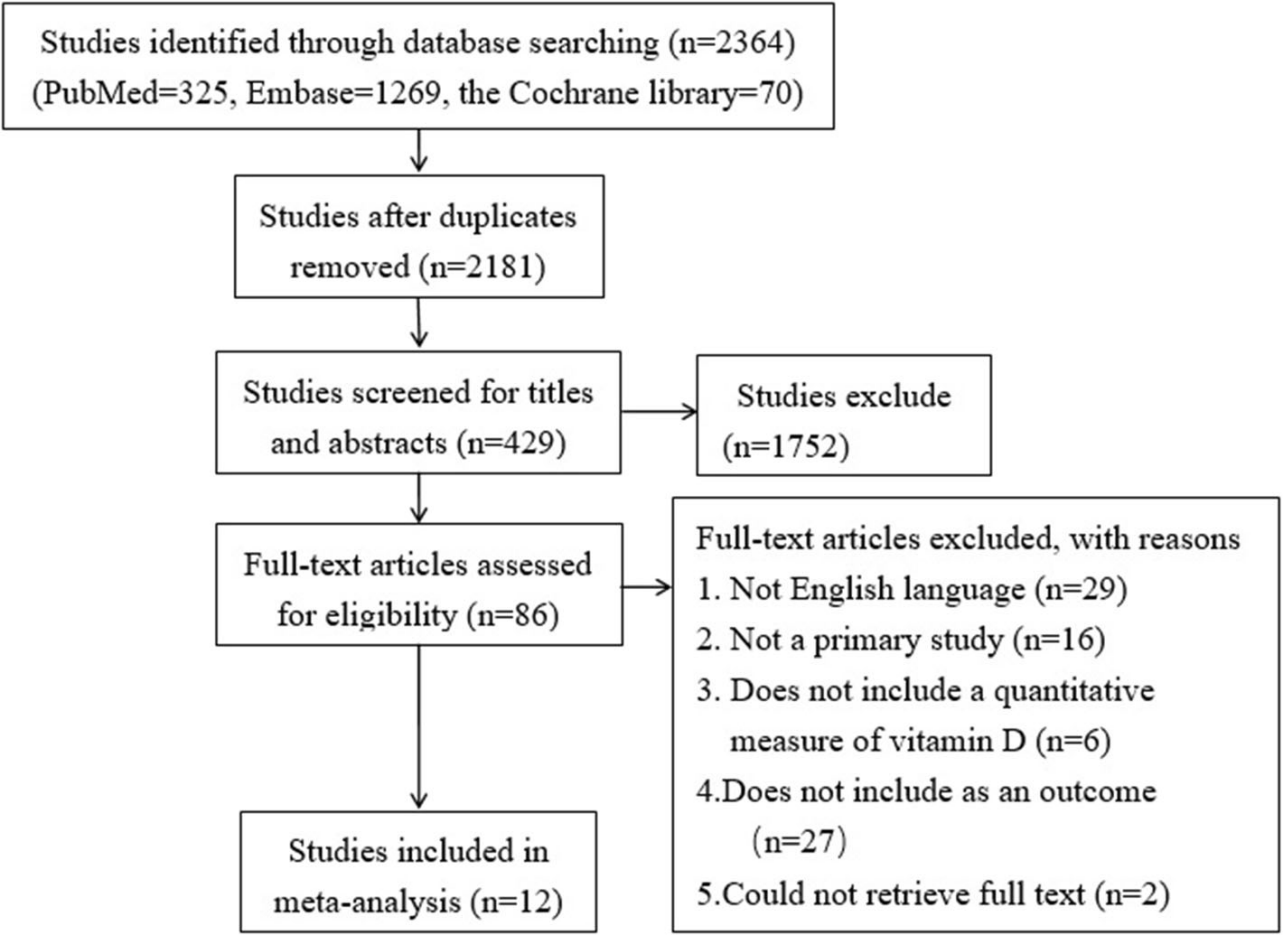
Flowchart of selection of studies for inclusion in the meta-analysis

The study screening flow chart is shown in Fig. 1. Overall, the systematic searched identified 2364 potentially relevant studies, of which 183 were duplicate studies and another 1752 were irrelevant and were thus excluded during the initial screening of the title and abstract. The studies included both sexes, except one which included only men [19]. There were eight prospective cohort studies [19–26] and four cross-sectional studies [27–30]. The studies were published between 2010 and 2018 (Table 1 and Table 2). Included studies originated from Denmark, the US, Finland, France, Sweden, Germany, and the Netherlands. Review articles, studies with duplicate data, and studies not published in English were all excluded. Quality assessments indicated that all included studies were of high quality.

**Table 1:** Summary characteristics of studies included in the analysis of vitamin D deficiency and risk of AD

Author, Publication year& country	Study Type	Sex	Age (Mean)	No.Patients (totle)	Follow-up duration (year)	25(OH)D (ng/ml)	OR	95% CI	Quality score	Vitamin D assessment method	Adjustment
Karakis, 2016, US	Prospective cohort	W/M	72.4	1663	9	< 10	0.97	0.47–2.00	8	Competitive protein-binding assay and radioimmunoassay	Age, gender, smoking, HTN, DM, prevalent CVD, homocysteine, BMI, and vitamin D supplement use.
Afzal, 2014, Denmark	Prospective cohort	W/M	58.0	4087	21	< 10 10–20	1.25 1.12	0.95–1.64 0.90–1.40	9	Not report	Age, sex, month of blood sample, smoking status, body mass index, leisure time and work-related physical activity, alcohol consumption, income level, education, baseline diabetes mellitus, hypertension, cholesterol, high-density lipoprotein cholesterol, and creatinine
Littlejohns 2014, US	Prospective Cohort	W/M	73.6	1547	5.6	< 10 10–20	2.22 1.69	1.02–4.83 1.06–2.69	8	Liquid chromatography tandemmass spectrometry (LC-MS)	Age, season of vitamin D collection, education, sex, BMI, smoking, alcohol consumption, and depressive symptoms
Feart, 2017, France	Prospective cohort	W/M	73.3	916	11.4	< 10 10–20	2.21 1.65	1.28–3.80 0.98–2.77	8	One-step immunoassay	Gender, education, income, depressive symptomatology, number of drugs per day, apolipoprotein E e4 allele, BMI, practice of physical exercise, DM, history of CVD and stroke, HTN, hypercholesterolemia, hypertriglyceridemia, smoking status, and Mediterranean diet score
Buell, 2010, US	Cross-sectional	W/M	73.5	318		< 20	2.65	0.99–7.16	7	Radioimmunoassay (DiaSorin, Inc.,Stillwater, MN, USA) on fasting blood sample	Age, race, sex, body mass index, and education, kidney function, multivitamin use, season, diabetes, hypertension, plasma homocysteine, and ApoE allele status
Licher, 2017, Netherlands	Prospective cohort	W/M	69.2	6087	13.3	< 20	1.10	1.00–1.19	9	Electrochemilumin-escence binding assay	Age, sex, season of blood collection, BMI, SBP, DBP, educational level, smoking, alcohol use, calcium serum levels, ethnicity, eGFR, TC, HDL, history of DM, HF, stroke, MI, depressive symptoms, outdoor activity, and APOE −4 carrier status.

**Table 2:** Summary characteristics of studies included in the analysis of vitamin D deficiency and risk of dementia

Author& Publication year	Study Type	Sex	Age (Mean)	No.Patients (totle)	Follow-up duration (year)	25(OH)D (ng/m)	OR	95% CI	Quality score	Vitamin D assessment method	Adjustment
Karakis, 2016, US	Prospective cohort	W/M	72.4	1663	9	< 10	1.06	0.57–1.98	8	Competitive protein-binding assay and radioimmunoassay	Age, gender, smoking, HTN, DM, prevalent CVD, homocysteine, BMI, and vitamin D supplement use.
Knekt, 2014, Finland	Prospective cohort	W/M	56.4	5010	17	< 10	1.74	0.64–3.01	8	Radioimmunoassay	Age, month of blood drawn, education, marital status, physical activity, smoking status, alcohol consumption, BMI, BP, FPG, serum TG, and serum TC.
Licher, 2017, Netherlands	Prospective cohort	W/M	69.2	6087	13.3	< 10 10–20	1.22 1.06	0.97–1.52 0.90–1.26	9	Electrochemiluminescence binding assay	Age, sex, season of blood collection, BMI, SBP, DBP, educational level, smoking, alcohol use, calcium serum levels, ethnicity, eGFR, TC, HDL, history of DM, HF, stroke, MI, depressive symptoms, outdoor activity, and APOE-4 carrier status.
Schneider, 2014, US	Prospective cohort	W/M	62.0	1652	16.6	< 10 10–20	1.53 1.22	0.84–2.79 0.68–2.19	8	Liquid chromatography-tandem mass spectrometry	Age, sex, education, income, physical activity, smoking, alcohol use, BMI, WC, and vitamin D supplementation.
Feart,2017, France	Prospective cohort	W/M	73.3	916	11.4	< 10 ng/ml 10–20	2.96	1.43–6.11	8	One-step immunoassay	Gender, education, income,
						ng/ml	2.29	1.14–4.58			depressive symptomatology, number of drugs per day, apolipoprotein E e4 allele, BMI, practice of physical exercise, DM, history of CVD and stroke, HTN, hypercholesterolemia, hypertriglyceridemia, smoking status, and Mediterranean diet score
Olsson, 2017, Sweden	Prospective Cohort	M	71.0	1182	12	< 10 10–20	1.22 1.06	0.97–1.52 0.90–1.26	8	HPLC atmospheric pressure chemical ionization-mass spectrometry	Age, season of blood collection, BMI, education, physical activity, smoking, DM, HTN, hypercholesterolemia, use of vitamin D supplements, and alcohol intake.
Littlejohns, 2014,US	Prospective Cohort	W/M	73.6	1615	5.6	< 10 10–20	2.25 1.53	1.23–4.13 1.06–2.21	9	Liquid chromatography tandemmass spectrometry (LC-MS)	Age, season of vitamin D collection, education, sex, BMI, smoking, alcohol consumption, and depressive symptoms
Annweiler, 2011, France	Cross-sectional	W/M	86.0	288	Not report	< 10	2.57	1.05–6.27	7	Radioimmunoassay (DiaSorin, Inc.,Stillwat er, MN,USA) on fastingblood sample	Fully adjusted but without detailed information
Nagel, 2015, Germany	Cross-sectional	M/W	75.6	1373	Not report	< 20	1.08	1.06–2.21	8	ELISA(ImmunodiagnosticSystems Inc., Fountain Hills, AZ,USA)	Adjusted for age, sex, school education, smoking status, season, alcohol consumption, BMI, and history of depression
Buell, 2010, USA	Cross-sectional	M/W	73.5	318	Not report	< 20	2.21	1.13–4.32	7	Radioimmunoassay (DiaSorin, Inc., Stillwater, MN,USA) on fasting blood sample	Age, race, sex, body mass index, and education, kidney function, multivitamin use, season, diabetes, hypertension, plasma homocysteine, and ApoE allele status
Nourhashemi,2018,French	Cross –sectional	M/W	76.2	1680	Not report	< 20	1.038	0.421–2.557	9	a commercially available electro-chemiluminescencecompetitive binding assay	gender, BMI, season of blood collection, educational level, and ApoE ε4 genotype

### Vitamin D status and risk of dementia

**Fig. 2:**
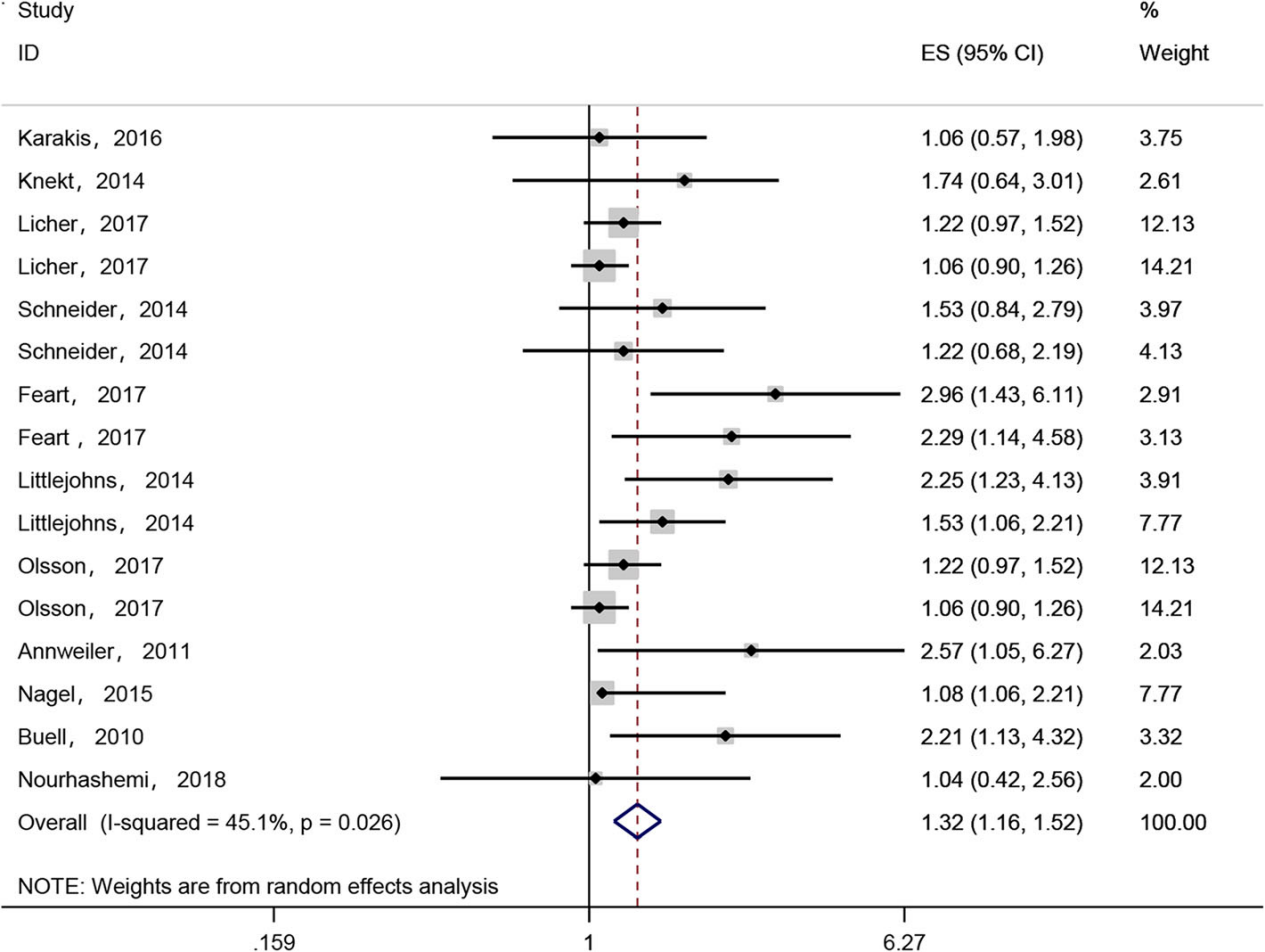
HRs of association between dementia and vitamin D deficiency (serum 25(OH)D < 20 ng/ml)**.** The size of each square is proportional to the study’s weight. The estimated pooled HR was 1.32 (95%CI, 1.16 to 1.52) with high statistical significance (*P* < 0.0001). There was moderate heterogeneity among the studies (*I*^*2*^ = 45.1%)

Eleven studies [18–24, 26–29] involving a total of 21,784 participants evaluated the relationship between vitamin D deficiency and dementia. We conducted a meta-analysis (random effects model) to derive a pooled estimate of the association between vitamin D deficiency (< 20 ng/ml) and the risk of dementia. The pooled HR estimate of the risk of dementia for those with vitamin D deficiency (< 20 ng/ml) relative to those with vitamin D levels in the normal range (≥20 ng/ml) was 1.32 (95% CI: 1.16, 1.52, *I*^*2*^ = 45.1%, *n* = 16, Fig. 2). Severe vitamin D deficiency (< 10 ng/ml) was associated with a high risk of dementia after pooling the results of the subgroup analysis (pooled HR: 1.48, 95%CI: 1.19, 1.85, *I* ^*2*^ = 41.4%, *n* = 8, Additional file 1: Fig. S4). However, the pooled results for moderate vitamin D deficiency (10–20 ng/ml) showed little association with dementia (pooled HR: 1.20, 95%CI: 0.99, 1.44, *I*^*2*^ = 48.3%, *n* = 5, Additional file 1: Fig. S4). The *I*^*2*^ value between 25 and 50% indicates evidence of moderate heterogeneity among the studies. In the subgroup analyses, a more significant association between vitamin D deficiency and dementia was observed in the subgroup of studies that considered the serum 25(OH)D APOE gene compared to studies that did not consider the APOE gene (pooled HRs: 1.47, 95%CI: 1.10, 1.98, *I*^*2*^ = 65.9%, *n* = 6 studies vs. 1.28, 95%CI: 1.10, 1.49, *I* ^*2*^ = 29.1%, *n* = 10 studies, respectively, Additional file 1: Fig. S6). Similarly, a more significant association was observed in cohort studies compared to cross-sectional studies (pooled HRs: 1.30, 95%CI: 1.13, 1.50, *I* ^*2*^ = 47.5%, *n* = 12 vs. 1.32, 95%CI: 1.16, 1.52, *I*^*2*^ = 48.4%, *n* = 4, respectively, Additional file 1: Fig. S8). These subgroup differences appear to be the sources of heterogeneity among the pooled studies. Moreover, meta-regression analysis showed a correlation between dementia risk and follow-up time (*P* = 0.03; β = 0.90, 95% CI, 0.88 to 1.01). This suggests that follow-up time could account for 90% of the heterogeneity among the studies. When removing each study in turn, the association between vitamin D deficiency and dementia ranged from 1.27 (95%CI: 1.12, 1.43) with the exclusion of the prospective cohort study by Littlejohns et al. [24] to 1.38 (95%CI: 1.19, 1.61) with the exclusion of the cross-sectional study by Annweiler et al. [26] (Additional file 1: Table S3). The sensitivity analysis indicated that omission of any one of the studies did not alter the magnitude of the observed effect, suggesting stability of our findings.

### Vitamin D status and risk of AD

**Fig. 3:**
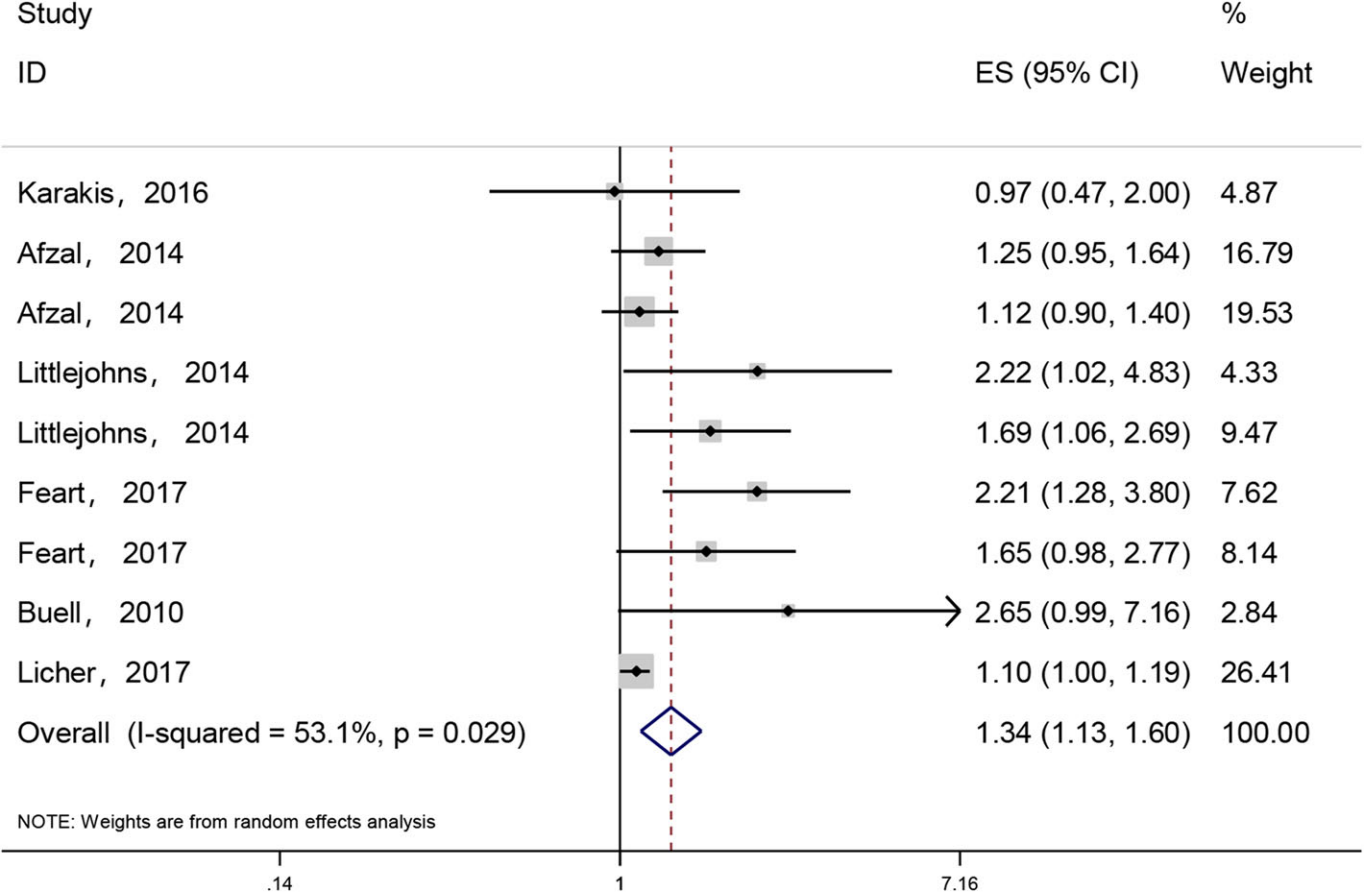
HRs of association between AD and vitamin D deficiency (serum 25(OH)D < 20 ng/ml). The size of each square is proportional to the study’s weight. The estimated pooled HR was 1.34 (95%CI, 1.13 to 1.60) with high statistical significance (*P* < 0.0001). There was high heterogeneity among the studies (*I*^*2*^ = 53.1%)

Six studies [19, 21, 23–25, 28] involving a total of 14,618 participants evaluated the relationship between vitamin D deficiency and AD. We performed a meta-analysis (random effects model) to derive a summary estimate of AD risk associated with vitamin D deficiency. The meta-analysis indicated that patients with vitamin D deficiency (< 20 ng/ml) had a higher risk of AD than patients with adequate vitamin D supply (≥20 ng/ml) (pooled HR: 1.34, 95% CI: 1.13, 1.60, *I*^*2*^ = 53.1%, *n* = 9, Fig. 3). The *I*^*2*^ value was greater than 50%, indicating evidence of heterogeneity among the studies. Severe vitamin D deficiency(< 10 ng/ml) was associated with a high risk of AD compared to moderate vitamin D deficiency (10–20 ng/ml) after pooling the results in the subgroup analysis (pooled HRs: 1.51, 95%CI: 1.04, 2.18, *I*^*2*^ = 47.4%, *n* = 4 vs. 1.36, 95%CI: 1.01, 1.84, *I*^*2*^ = 39.6%, *n* = 3, Additional file 1: Fig. S5). The *I*^*2*^ value between 25 and 50% indicates moderate heterogeneity among the studies. In the subgroup analyses, a more significant association between vitamin D deficiency and AD was observed in the subgroup of studies that considered the serum 25(OH)D APOE gene compared to studies that did not consider the APOE gene (pooled HRs: 1.34, 95%CI: 1.13, 1.60, *I*^*2*^ = 72.9%, *n* = 5 vs. 1.27, 95%CI:1.05, 1.54, *I*^*2*^ = 28.1, n = 4, respectively). Moreover, meta-regression analysis indicated an association between dementia risk and follow-up time (*P* = 0.02; β = 0.89, 95% CI, 0.87 to 1.06). 11This suggests that follow-up time could account for 89% of the heterogeneity among the studies. In the sensitivity analysis, with removal of each study in turn, the association between vitamin D deficiency and AD ranged from 1.13 (95%CI: 1.10, 1.55) with the exclusion of the cross-sectional study by Bull et al. [28] to 1.46 (95%CI: 1.18, 1.80) with the exclusion of the prospective cohort study by Licher [21] (Additional file 1: Table S4). As a result of the sensitivity analysis, one of the studies[28]was found to be the heterogeneous source of this review after each study was excluded in sequence.

## Publishing bias

**Fig. 4:**
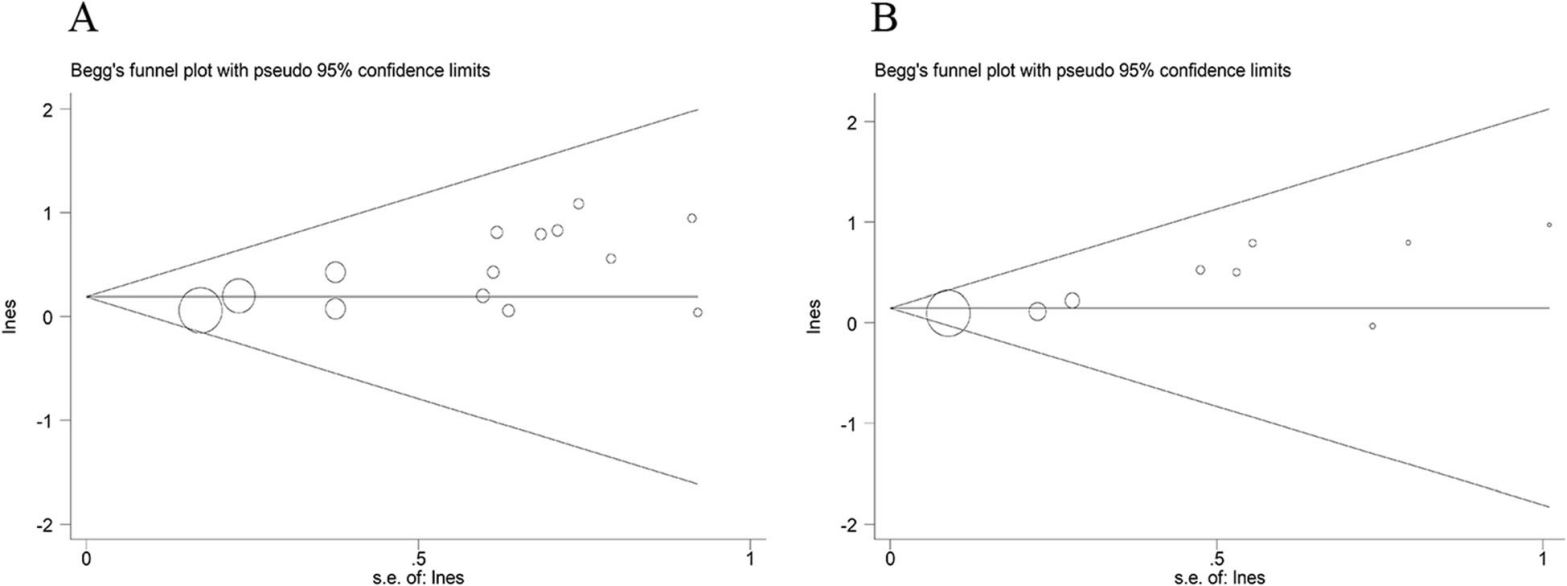
Publication bias for studies examining the associations between vitamin D deficiency (serum 25(OH)D < 20 ng/ml) and dementia and AD**.** The size of each circle is proportional to the study’s weight. The Begg’s tests for the incidence of dementia (A) and AD (B) were roughly symmetrical (*P*_(dementia)_ = 0.061, P_(AD)_ = 0.076, greater than 0.05)

The funnel plot was symmetrical indicating no evidence of publication bias (Fig. 4).

## Discussion

The current meta-analysis provided additional evidence of relationships between vitamin D deficiency and risk of dementia and AD. The present analysis demonstrated positive associations between vitamin D deficiency (< 20 ng/ml) and risk of dementia (by 32%) and AD (by 34%). These results demonstrated that vitamin D deficiency may be a risk factor for dementia or AD. Furthermore, the incidence of AD was higher than dementia when vitamin D was deficient. The subgroup analyses indicated that vitamin D severe deficiency was associated with a greater risk of dementia (by 48%) and AD (by 51%) compared to vitamin D moderate deficiency (by 20 and 36%, respectively), indicating that the risk of dementia and AD was reduced with increased vitamin D. It also suggested that the incidence of AD may be higher than dementia in either mild or moderate vitamin D deficiency. In addition, dementia-related genes and several confounding factors were potential sources of the heterogeneity among the studies. The subgroup of considering the APOE gene was more heterogeneous than the without considering APOE gene. This may reflect different study quality or selectiveness of recruitment. Meta-regression showed that follow-up time may be a source of heterogeneity, which may be related to inconsistencies in the follow-up time of each included study. One study included only male cases and may be a source of potential heterogeneity. A recent review proposed calcium homeostasis as the underlying factor, suggesting that long-term deficiency or inefficient utilization of vitamin D may lead to disruption of calcium homeostasis in neurons, thus causing neuronal aging and neurodegeneration vulnerability [31]. The most common causes of dementia include Lewy body dementia, depression, Alzheimer’s disease, and mild cognitive impairment. Other dementias are less common. AD is the most common neurodegenerative disorder in older people and leads to progressive cognitive impairment. Dementia and AD lead to decreased function and dependence on others for daily life activities; this places a significant financial burden on the health care system. Recent data suggests a possible association between vitamin D deficiency and cognitive dysfunction [32]. Current studies have not fully established the pathophysiological mechanisms underlying the potential effects of vitamin D levels on dementia and AD risk, but several possible mechanisms have been proposed. The potential pathological mechanisms that have been identified to date and that are used to diagnose clinical cognitive dysfunction include Aβ deposition and tau protein tangles in the brain [33]. Animal studies have shown that vitamin D can promote the clearance of Aβ and vitamin D deficiency leads to an increase in Aβ in the brain [34]. In human studies, it has been shown that vitamin D causes an increase in plasma Aβ, especially in the elderly, suggesting a decrease in brain Aβ [35]. In addition, vitamin D modulates the voltage-gated calcium channel targeted by Aβ peptides, indicating that vitamin D can repair neuronal calcium homeostasis altered by Aβ peptides [36]. Finally, vitamin D can prevent glutamate neurotoxicity by upregulating VDR expression and exerting antioxidant effects [12]. AD is a degenerative disease characterized by cognitive and functional decline. Due to their loss of autonomy, it is difficult for patients to receive enough sunlight exposure in order to synthesize a sufficient amount of vitamin D. Similarly, it can be difficult for these patients to eat a sufficient amount of foods rich in vitamin D [37]. This exogenous vitamin D restriction could subsequently lead to low serum vitamin D concentrations in AD patients. Compared with previous reviews, we considered the relationships between different levels of vitamin D and both dementia and AD, and also considered the effects of dementia-related genes and follow-up duration. Compared with previous reviews, our meta-analysis included recently published studies and contained sufficient data for subgroup analysis and exploration of various associations. Thus, this study can provide comprehensive and systematic evidence for clinical adjuvant treatment of dementia and AD. Furthermore, due to small numbers of included studies (< 10) and failure to investigate publication bias, previous reviews may have overestimated the associations between vitamin D deficiency and risk of dementia and AD. Previous meta-analyses have shown that the measures used to assess the relationship between vitamin D and dementia are relatively simple. This may create a bias in the literature as the measures used in the published literature cover less than 30% of the available neuropsychological tests [38]. When all neuropsychological tests were included, despite the increased heterogeneity, we found a significant association between vitamin D deficiency and dementia. In recent years, genes associated with dementia and AD, such as APOE, have received increased research attention. As such, we considered the possible role of genetic factors in this meta-analysis [39].The overall effect size should be interpreted with caution due to the presence of known or unknown confounding factors. The use of a random effects meta-analysis model in this study compensated for the heterogeneity among the studies. It should also be noted that heterogeneity was markedly reduced in our analysis as compared to previous studies; this could reflect the more stringent inclusion criteria. Finally, the significant pooled effect in the current study suggests that these methodological factors were advantageous in our meta-analysis, conferring greater clinical applicability of the findings and greater generalizability of the association between vitamin D deficiency and dementia. When interpreting the current results, the heterogeneity among the included studies should be considered. First, none of the included studies described the methods of collection or duration of preservation of collected serum before 25(OH)D assay. The effects of light, temperature, collection tube properties, and long-term serum storage on assay results are uncertain, particularly with respect to 25(OH)D stability [40]. Second, the methods for determining the concentration of 25(OH)D among the studies were different. The choice of method is crucial since IDEQAS reported differences in results for identical blood samples as a function of method chosen. Therefore, harmonization of techniques seems to be necessary. The most commonly used vitamin D reference assay is the LC-MS assay [41]. However, even inter-laboratory variability in measurements with this technique is unavoidable. Further, since conventional LC-MS analysis may not be able to distinguish molecules of the same mass as 25(OH)D_3_ and may not be able to distinguish among molecules with similar fragmentation patterns, the concentration of 25(OH)D is often overestimated [41]. In contrast, radioimmunoassay, which is a better analytical method than LC-MS, exhibits good inter-rater reliability and can determine the concentrations of 25(OH)D_2_ and 25(OH)D_3_ simultaneously [42]. It appears to be the most economical technology for standardization in future research [43]. Third, none of the included studies tested available vitamin D; however, several studies have shown that available vitamin D may be a more reliable marker of vitamin D status than the total amount of 25(OH)D [44]. Fourth, vitamin D needs to be metabolized by the kidneys to produce active 1,25-(OH)_2_D_3_; thus, chronic kidney disease may affect the metabolism of vitamin D. However, only one study considered chronic kidney disease in the multivariate analysis [28]. Fifth, the confounding influence of homocysteine was only adjusted for in a few of the included studies [19, 28]. Excessive homocysteine increases the risk of endothelial dysfunction and increases the cardiovascular risk, which directly contributes to the development of dementia and AD by promoting neurodegenerative changes [45]. Therefore, failure to adjust for this confounding factor may result in biased conclusions. Finally, in the present review, because cross-sectional studies are affected by reverse causality bias, it is not possible to determine whether low levels of vitamin D cause dementia. Instead, dementia may result in reduced intake of vitamin D and reduced outdoor activity with impaired sunlight exposure, both leading to vitamin D deficiency [37]. Several of the included studies improve the reliability of the results by adjusting for two confounding factors, namely, physical activity level and season of sampling [18, 20, 21, 23–25, 27, 28]. In summary, the results of this meta-analysis should be interpreted with caution due to the methodological differences and the confounding factors among the included studies. In the early stages of dementia, for example, mild cognitive impairment is more likely to benefit from therapeutic intervention; thus, more data on the association between mild cognitive impairment and vitamin D would be helpful [46]. Therefore, future prospective studies should examine the association between vitamin D deficiency and the early stages of AD and dementia (e.g., mild cognitive impairment).

## Conclusions

The current meta-analysis provided additional evidence of the relationships between vitamin D deficiency (< 20 ng/ml) and risk of dementia and AD in older age people. This study found significant positive associations between vitamin D deficiency (< 20 ng/ml) and risk of dementia and AD. Subgroup analysis indicated that vitamin D moderate deficiency (< 10 ng/ml) was more strongly associated with the risk of dementia and AD compared with severe deficiency (10–20 ng/ml), indicating that the risk of dementia and AD was reduced with increased vitamin D. However, we have no clear evidence of an association between serum 25(OH)D levels > 20 ng/ml and cognitive outcomes. Further longitudinal investigations are needed to assess cognitive outcomes at higher 25(OH)D levels.

## Supplementary information

**Additional file 1: Appendix 1.** Included search strategies, sensitivity analysis tables, and subgroup analyses **Appendix 2.** Sensitivity analysis supplementary table for systematic reviews. **Table S3.** Sensitivity analysis of AD associated with vitamin D Deficiency (serum 25(OH)D < 20 ng/ml). **Table S4** Sensitivity analysis of AD associated with vitamin D Deficiency (serum 25(OH)D < 20 ng/ml). **Appendix 3.** Sensitivity analysis supplementary table for systematic reviews**. Figure S4.** HRs of AD associated with different levels of vitamin D deficiency. **Figure S5.** HRs of AD associated with different levels of vitamin D deficiency. **Figure S6.** HRs of dementia that considered the APOE gene of serum 25(OH)D compared to without considering the APOE gene. **Figure S7.** HRs of AD that considered the APOE gene of serum 25(OH)D compared to without considering the APOE gene. **Figure S8.** HRs of dementia associated with vitamin D Deficiency that cohort studies compared to cross-sectional studies.

## Data Availability

All data analyzed during this study are included in this article.

## Abbreviations

25(OH)D: 25-dihydroxyvitamin D
7-DHC: 7-dehydrocholesterol
AD: Alzheimer’s disease
APOE: Apolipoprotein E
Aβ: beta-amyloid
BMI: Body mass index
CI: Confidence interval
CI: Confidence interval
ELISA: Enzyme linked immunosorbent assay
HR: hazard ratio
IDEQAS: International Vitamin D External Quality Assessment Scheme
LC-MS: liquid chromatography-tandem mass spectrometry
NOS: Newcastle-Ottawa Scale
PICO: Patient intervention comparison outcome
US: United States
VDR: vitamin D recepter
