# Supplementary material for: Vitamin D deficiency as a risk factor for dementia and Alzheimer’s disease: an updated meta-analysis
Source: BMC Neurol. 2019 Nov 13;19:284. doi: 10.1186/s12883-019-1500-6 (PMC6854782; doi:10.1186/s12883-019-1500-6)
Supplement: Supplementary file 1 — Additional file 1: Appendix 1. Included search strategies, sensitivity analysis tables, and subgroup analyses Appendix 2. Sensitivity analysis supplementary table for systematic reviews. Table S3. Sensitivity analysis of AD associated with vitamin D Deficiency (serum 25(OH)D < 20 ng/ml). Table S4 Sensitivity analysis of AD associated with vitamin D Deficiency (serum 25(OH)D < 20 ng/ml). Appendix 3. Sensitivity analysis supplementary table for systematic reviews. Figure S4. HRs of AD associated with different levels of vitamin D deficiency. Figure S5. HRs of AD associated with different levels of vitamin D deficiency. Figure S6. HRs of dementia that considered the APOE gene of serum 25(OH)D compared to without considering the APOE gene. Figure S7. HRs of AD that considered the APOE gene of serum 25(OH)D compared to without considering the APOE gene. Figure S8. HRs of dementia associated with vitamin D Deficiency that cohort studies compared to cross-sectional studies. [file 12883_2019_1500_MOESM1_ESM.doc]

### Appendix 1. search strategy for systematic review

The database of **Pubmed** strategy

#1 "Dementia"[Mesh]

#2 "dement*"[tw]

#3 "lewy body disease"[tw]

#4 "huntington disease"[tw]

#5 "kluver Bucy syndrome"[tw]

#6 "rett syndrome"[tw]

#7 "frontotemporal dementia"[tw]

#8 "vascular dementia"[tw]

#9 "Alzheimer Disease"[Mesh]

#10 "alzheimer's disease"[tw]

#11 "alzheimer"[tw]

#12 "AD"[tw]

#13 "Vitamin D Deficiency"[Mesh]

#14 "vitamin d"[tw]

#15 "Vitamin D Deficiency"[tw]

#16 "25 hydroxyvitamin D"[tw]

#17 "25(OH)D"[tw]

#18 "humans"[mh]NOT"animals "[mh]

**#19** #1OR#2OR#3OR#4OR#5OR#6OR#7#8

**#20** #9OR#10OR#11OR#12

**#21** #13OR#14OR#15OR#16OR#17

**#22** #18AND#19AND#20AND#21

The database of **the Chochrane library** strategy

#1 MeSH descriptor: [Dementia] explode all trees

#2 (Dementia):ti,ab,kw(Word variations have searched)

#3 (Lewy Body Disease):ti,ab,kw(Word variations have searched)

#4 (Frontotemporal Lobar Degeneration):ti,ab,kw(Word variations have searched)

#5 (Huntington Disease):ti,ab,kw(Word variations have searched)

#6 (Kluver-Bucy Syndrome):ti,ab,kw(Word variations have searched)

#7 (Creutzfeldt-Jakob Syndrome):ti,ab,kw(Word variations have searched)

#8 (vascular dementia) :ti,ab,kw(Word variations have searched)

#9 MeSH descriptor: [Alzheimer Disease] explode all trees

#10 (Alzheimer's disease):ti,ab,kw(Word variations have searched)

#11 MeSH descriptor: [Vitamin D] explode all trees

#12 (Vitamin D):ti,ab,kw(Word variations have searched)

#13 (25-Hydroxyvitamin D):ti,ab,kw(Word variations have searched)

#14 {OR #1-#8}

#15 {OR #9-#10}

#16 {OR #11-#13}

#17{AND #14-#16}

The database of **Embase** strategy

#1 'dementia'/exp

#2 'dement*'

#3 'lewy body disease'

#4 'frontotemporal dementia'

#5 'huntington chorea'

#6 'rett syndrome'

#7 'kluver Bucy syndrome'

#8 'vascular dementia'

#9 'alzheimer disease'/exp

#10 'alzheimer's disease'

#11 'alzheimer'

#12 'AD'

#13 'vitamin d'/exp

#14 'vitamin d'

#15 '25 hydroxyvitamin D'/exp

#16 '25 hydroxyvitamin D'

#17 '25(OH)D'

**#18** #1OR#2OR#3OR#4OR#5OR#6OR#7#8

**#19** #9OR#9OR#10OR#12

**#20** #13OR#13OR#14OR#15OR#17

**#21** #18AND#19AND#20

The search strategy detailed lists

("vitamin D" OR "25(OH)D" OR "25 hydroxyvitamin D" OR "Vitamin D Deficiency") AND ("Alzheimer's disease" OR "dementia" OR "dement*" OR "lewy body disease" OR "huntington disease" OR "huntington chorea" OR " Frontotemporal Lobar Degeneration " OR "kluver Bucy syndrome" OR "rett syndrome" OR "frontotemporal dementia" OR "vascular dementia" OR "Creutzfeldt-Jakob Syndrome" OR "alzheimer" OR "AD")

**Appendix 2.** Sensitivity analysis supplementary table for systematic reviews

| Exclusion study | ES | 95%CI | *I^2^(%)* |
| --- | --- | --- | --- |
| Nourhashemi， 2018 | 1.34 | (1.17,1.55) | 45.5 |
| Buell， 2010 | 1.32 | (1.15,1.51) | 47.1 |
| Nagel， 2015 | 1.36 | (1.17,1.59) | 48.8 |
| Annweiler， 2011 | 1.38 | (1.19,1.61) | 42.5 |
| Olsson， 2017 | 1.32 | (1.15,1.52) | 47.6 |
| Olsson， 2017 | 1.34 | (1.16,1.54) | 48.8 |
| Littlejohns， 2014 | 1.27 | (1.12,1.43) | 34.6 |
| Littlejohns， 2014 | 1.29 | （1.13,1.47） | 41.7 |
| Feart ，2017 | 1.28 | （1.13,1.46） | 39.6 |
| Feart， 2017 | 1.31 | （1.14,1.51） | 45.4 |
| Schneider，2014 | 1.36 | （1.17,1.59） | 48.8 |
| Schneider，2014 | 1.32 | （1.16,1.52） | 45.1 |
| Licher，2017 | 1.38 | （1.19,1.61） | 42.5 |
| Licher，2017 | 1.30 | （1.14,1.48） | 43.0 |
| Knekt，2014 | 1.29 | （1.13,1.47） | 42.1 |
| Karakis，2016 | 1.34 | （1.16,1.54） | 48.6 |

**Supplemental Table 3** Sensitivity analysis of AD associated with vitamin D Deficiency (serum 25(OH)D <20 ng/ml).

| Exclusion study | ES | 95%CI | *I^2^* |
| --- | --- | --- | --- |
| Karakis，2016 | 1.38 | （1.14,1.66） | 58.4 |
| Afzal， 2014 | 1.40 | （1.13,1.73） | 58.3 |
| Afzal， 2014 | 1.45 | （1.16,1.83） | 58.7 |
| Littlejohns， 2014 | 1.30 | （1.10,1.55） | 51.3 |
| Littlejohns， 2014 | 1.31 | （1.09,1.56） | 51.7 |
| Feart， 2017 | 1.25 | （1.08,1.46） | 39.6 |
| Feart， 2017 | 1.32 | （1.10,1.58） | 54.2 |
| Buell， 2010 | 1.13 | （1.10,1.55） | 51.3 |
| Licher，2017 | 1.46 | （1.18,1.80） | 40.7 |

**Supplemental Table 4** Sensitivity analysis of AD associated with vitamin D Deficiency (serum 25(OH)D <20 ng/ml).

**Appendix 3.** Sensitivity analysis supplementary table for systematic reviews

**Supplemental figure 4** HRs of dementia associated with different levels of vitamin D deficiency.

**Supplemental figure 5** HRs of AD associated with different levels of vitamin D deficiency.

**Supplemental figure 6** HRs of dementia that considered the APOE gene of serum 25(OH)D compared to without considering the APOE gene.

**Supplemental figure 7** HRs of AD that considered the APOE gene of serum 25(OH)D compared to without considering the APOE gene.

**Supplemental figure 8** HRs of dementia associated with vitamin D Deficiency that cohort studies compared to cross-sectional studies.
